# Focal distortion of the nuclear envelope by huntingtin aggregates revealed by lamin immunostaining

**DOI:** 10.1016/j.neulet.2008.09.075

**Published:** 2008-12-12

**Authors:** J. Paul Chapple, Virginie Bros-Facer, Rachel Butler, Jean-Marc Gallo

**Affiliations:** MRC Centre for Neurodegeneration Research, King’s College London, Department of Clinical Neuroscience, Institute of Psychiatry, De Crespigny Park, London SE5 8AF, United Kingdom

**Keywords:** Polyglutamine, Huntingtin, Aggregation, Lamin, Nuclear envelope

## Abstract

Huntington’s disease is an autosomal dominant neurodegenerative disorder caused by the expansion of a polyglutamine repeat tract in the huntingtin protein. Polyglutamine-expanded huntingtin forms intranuclear as well as perinuclear inclusion bodies. Perinuclear aggregates formed by polyglutamine-expanded proteins are associated with a characteristic indentation of the nuclear envelope. We examined the nuclear envelope in cells containing huntingtin aggregates using immunostaining for lamin B1, a major component of the nuclear lamina. Laser confocal microscopy analysis revealed that huntingtin aggregates in a juxtanuclear position were associated with a clear focal distortion in the nuclear envelope in cells transfected with polyglutamine-expanded huntingtin. Lamin B1 distribution was not altered by aggregates of polyglutamine-expanded ataxin-1, that are exclusively intranuclear. Thus lamin immunocytochemistry demonstrates clearly the depression of the nuclear envelope resulting from the formation of perinuclear aggregates by polyglutamine-expanded huntingtin. Lamin immunocytochemistry would be of value to monitor the state of the nuclear envelope in experimental paradigms aimed at establishing the significance of perinuclear aggregates of pathogenic proteins.

Huntington’s disease (HD) is an autosomal dominant neurodegenerative disorder characterized by selective neuronal loss in the striatum and cerebral cortex. HD is caused by the expansion of a polyglutamine (polyQ) repeat tract in the N-terminus of the huntingtin protein. HD is part of a group of inherited neurodegenerative diseases, that include HD, spinal and bulbar muscular atrophy and several spinocerebellar ataxias (SCA), caused by the expansion of a polymorphic CAG repeat domain in the open reading frame of the disease gene, translated into an expanded polyQ sequence in the gene product [11,17].

Long polyQ sequences in pathogenic proteins result in misfolding and propensity to aggregate into characteristic nuclear and cytoplasmic ubiquitinated inclusion bodies that are the most salient features of polyQ expansion disorders [5,6,9,16]. Whether aggregates of polyQ-expanded proteins are toxic or protective is still a matter of debate [13]. Huntingtin aggregation is a dynamic process and aggregates have been suggested to be toxic or cytoprotective, according to the stage of their formation [12] and the vulnerability of neurons to polyQ-expanded protein toxicity may be linked to their inability to sequester the protein [2,4].

In transfected cultured cells, inclusion bodies of polyQ-expanded proteins localize to the nucleus as well as to the cytoplasm where they can form perinuclear aggregates [22–24]. Electron microscopy studies have shown that perinuclear aggregates of huntingtin are associated with a typical distortion of the nuclear surface [23]. However, the state of the nuclear envelope adjacent to a huntingtin aggregate has received little attention. The nuclear envelope plays an important role in cellular organization [8] and disruption of the nuclear envelope in HD would be significant in the pathogenic process. For a global assessment of the state of the nuclear envelope, we examined the nuclear lamina in cells containing perinuclear aggregates formed by pathogenic polyQ-expanded huntingtin.

Chinese hamster ovary (CHO) cells were maintained in a humidified atmosphere of 95% air/5% CO_2_ at 37 °C in DMEM supplemented with 10% (v/v) heat inactivated fetal bovine serum (FBS, Invitrogen), 100 U/ml penicillin/100 μg/ml streptomycin and 2 mM l-glutamine. For transfection, cells were grown to 60–70% confluence in 6-well plates. Cells were transfected with EGFP-tagged huntingtin exon 1 containing 103 glutamines (Htt103Q) [25]. Cells were transfected with 3 μg DNA per well using Lipofectamine Plus reagent (Invitrogen). Cells were exposed to the DNA/liposome complex for 5 h in Optimem (Invitrogen) before being returned to normal growth medium. Cells were fixed in methanol at −20 °C for 5 min 24 or 48 h after transfection. Cells were then processed for immunofluorescence microscopy to visualize the nuclear lamina. The nuclear lamina, on the inner surface of the nuclear envelope, is composed of the type V intermediate filament proteins, lamins [19]. For a global assessment of the state of the nuclear envelope, we analyzed the distribution of lamin B1. Lamin B1 was chosen as, unlike lamin A/C which provides mechanical strength, lamin B is an indicator of nuclear envelope integrity [15]. Fixed cells were blocked in PBS containing 3% (w/v) bovine serum albumin and 10% (v/v) normal donkey serum for 30 min at room temperature and incubated with a monoclonal antibody to lamin B1 (L-5, Zymed) followed by Cy3-conjugated donkey anti-mouse antibody (Jackson ImmunoResearch Laboratories). Between incubations, cells were washed in PBS over 20 min. After processing coverslips were mounted in Vectashield (Vector Laboratories). Cells were imaged using a Zeiss LSM510 META laser scanning confocal microscope.

N-terminal fragments of huntingtin with a number of glutamine repeats in the normal range (i.e. <35) has a diffuse distribution in transfected cells; by contrast, huntingtin with a polyQ sequence over 35 repeats forms characteristic inclusions [6,25]. In CHO cells, Htt103Q aggregates have perinuclear or cytoplasmic (Fig. 1A and C) as well as nuclear (Fig. 2A and C) localizations. The nuclear lamina of cells not expressing Htt103Q appeared as a well-defined, lamin B1 positive, outline (Fig. 1B). By contrast, in cells expressing Htt103Q, lamin B1 staining revealed that huntingtin aggregates in a juxtanuclear position were associated with a clear focal distortion in the nuclear envelope (Fig. 1B and C). Although aggregates exert a mechanical pressure on the nuclear envelope, there were no overtly apparent discontinuity in the nuclear rim that would have been indicative of a local disruption of the nuclear envelope. No abnormality of the nuclear envelope was observed in cells with cytoplasmic (Fig. 1B and C) or nuclear (Fig. 2) aggregates.

We also examined the distribution of lamin B1 in cells containing aggregates of polyQ-expanded ataxin-1, the product of the SCA1 gene. CHO cells were transfected with EGFP-tagged ataxin-1 containing 82 glutamines (Atxn82Q) [7] and processed for lamin B1 immunostaining as described for huntingtin-transfected cells. Unlike huntingtin, aggregates formed by Atxn82Q are exclusively nuclear in transfected cells ([20] and Fig. 3A and C). Some Atxn82Q is observed in the perinuclear region of the cytoplasm of transfected cells, but appears as amorphous material not forming defined inclusions as seen in the nucleus (Fig. 3A and C). Confocal imaging of Atxn82Q-transfected cells immunostained for lamin B1 failed to reveal any abnormality of the nuclear envelope nor lamin B1 redistribution (Fig. 3B and C).

Lamin staining demonstrates clearly the depression of the nuclear envelope adjacent to a perinuclear aggregate of polyQ-expanded huntingtin. Alterations of nuclear envelope components have been associated with some neurological diseases. For example, a form of the hereditary motor and sensory neuropathy, Charcot-Marie-Tooth disease, type 2 (CMT2), is caused by mutations in the *LMNA* gene, encoding lamin A [10]. Intranuclear inclusions present in neurons of patients with Fragile X Tremor Ataxia Syndrome have been shown to recruit lamin A [14]. Many proteins involved in neurodegenerative diseases aggregate into perinuclear structures, including proteins associated with Lewy bodies in Parkinson’s disease [1,3,21] and retinitis pigmentosa rhodopsin mutants [18]. Lamin immunocytochemisty would represent a method of choice to monitor the state of the nuclear envelope in experimental paradigms aimed at establishing the pathogenic significance of protein aggregation.

## Figures and Tables

**Fig. 1:**
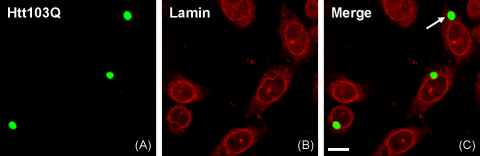
Confocal microscopy analysis of the nuclear lamina in polyQ-expanded huntingtin-expressing cells. CHO cells were transfected with huntingtin exon 1 with 103 glutamines tagged with EGFP (Htt103Q). Cells were stained with an antibody to lamin B1 and imaged by laser scanning confocal microscopy. Perinuclear Htt103Q aggregates are associated with a distortion of the nuclear envelope, clearly evident with lamin B1 staining. By contrast, cells with cytoplasmic aggregates (arrows) have an intact nuclear envelope. (A) EGFP fluorescence, (B) lamin B1 staining, and (C) merged signals. Scale bar = 10 μm.

**Fig. 2:**
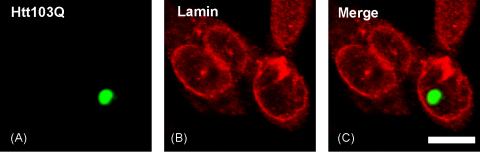
Intranuclear aggregates of polyQ-expanded huntingtin do not alter lamin B1 distribution in transfected cells. CHO cells were transfected with huntingtin exon 1 with 103 glutamines tagged with EGFP (Htt103Q) and stained for lamin B1. (A) EGFP fluorescence, (B) lamin B1 staining, and (C) merged signals. Scale bar = 10 μm.

**Fig. 3:**
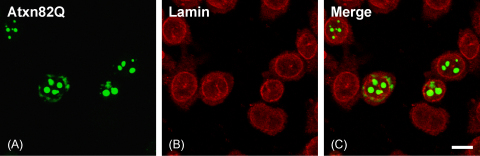
Nuclear aggregates of ataxin-1 do not alter lamin B1 distribution in transfected cells. CHO cells were transfected with EGFP-tagged ataxin-1 with 82 glutamines (Atxn82Q) and stained for lamin B1. Atxn82Q forms characteristic intranuclear aggregates but the nuclear envelope or the distribution of lamin B1 are not affected. (A) EGFP fluorescence, (B) lamin B1 staining, and (C) merged signals. Scale bar = 10 μm.
